# From food to vesicle: nutritional influences on gut microbial inflammatory signaling

**DOI:** 10.3389/fnut.2026.1756462

**Published:** 2026-02-02

**Authors:** Jari Verbunt, Lisa Mennens, Johan Jocken, Ellen E. Blaak, Paul Savelkoul, Frank R. M. Stassen

**Affiliations:** 1 Department of Medical Microbiology, Infectious Diseases & Infection Prevention, School of Nutrition and Translational Research in Metabolism (NUTRIM), Maastricht University Medical Center+, Maastricht, Netherlands; 2 Department of Human Biology, School of Nutrition and Translational Research in Metabolism (NUTRIM), Maastricht University Medical Center+, Maastricht, Netherlands; 3 Rehabilitation of Cardiorespiratory and Internal Diseases, Faculty of Rehabilitation and Physiotherapy (REVAL), Hasselt University, Hasselt, Belgium

**Keywords:** bacterial membrane vesicles, inflammation, microbiome, microbiota, modulation, nutrition

## Abstract

Diet is a pivotal determinant of gut microbial ecology, giving not only rise to specific bacterial compositionality but also its functional output. Studying functional readouts—such as microbial metabolite production—could provide a more accurate and mechanistically informative measure of intervention outcome than traditional compositional profiling alone. Bacterial membrane vesicles (bMVs) are gaining attention as mediators of microbial metabolism and output. These nanoparticles are selectively released as carriers of bioactive proteins, lipids, nucleic acids, and metabolites reflective of the activity of the parent bacteria. Importantly, bMVs are rigid, can efficiently be isolated from feces, and are able to stably transport their cargo to interact with the host. In interacting with immune cells or pathogen recognition receptors, they can potentiate inflammatory responses. Given their extensive, multifaceted involvement in inter-Kingdom communication, bMVs represent an important biomarker for evaluating dietary modulation of gut microbial function. We propose that characterization of gut-derived bMVs offers a highly sensitive, mechanistically grounded approach to titrating impact of dietary interventions. By capturing shifts in microbial metabolic activity and inflammatory potential, bMV-based assessments could complement or surpass traditional measures of microbiome compositional change. Integrating bMV profiling into dietary intervention studies may therefore provide new insight into the functional consequences of diet–microbiome interactions and help refine strategies aimed at reducing inflammation and promoting host health.

## Graphical abstract

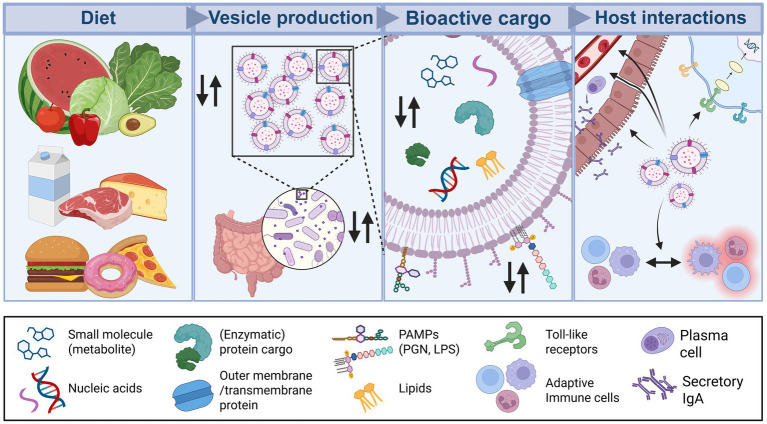

## Highlights

- Diet profoundly shapes and modulates the gut bacterial microbiome, and intervention outcome is best assessed by changes in functionality rather than compositionality.
- Bacterial membrane vesicles reflect bacterial functionality and metabolism, and are a potent inducer of inflammatory signaling.
- Effects of (dietary) interventions could be titrated by assessing gut-derived bacterial membrane vesicles.

## Introduction

The human gastrointestinal tract is colonized by approximately 10^13^–10^15^ bacteria that carry out essential functions in digestion, colony resistance and immune training (1). A disturbance in the composition or function of this microbiome is implicated in diseases including type 2 diabetes (T2D), obesity, inflammatory bowel disease (IBD), dementia and cancer (1, 2). For example, a colonic bloom of Pseudomonadota (Proteobacteria) is commonly reported in diseases such as T2D (3, 4) obesity (5), and IBD (6, 7). Reducing the abundance or activity of Pseudomonadota may help prevent disease onset, target its etiology, or mitigate resulting pathophysiological processes.

Strategies to modulate the activity of such intestinal pathobionts include dietary interventions, aimed at stimulating growth and activity of benign bacteria at cost of malignant ones. However, microbiota profiling techniques such as metagenomic or marker-gene sequencing (e.g., 16S ribosomal RNA or shotgun sequencing) reveal community composition but not microbial activity (8). For example, the presence of a bacterial toxin-encoding gene, does not guarantee that this gene is indeed expressed (9). For this reason, complementary omics approaches (e.g., metatranscriptomics, metaproteomics, metabolomics, and lipidomics) are becoming more central in microbiome research (10–13). Incorporating these techniques beyond DNA-based compositional profiling provides deeper insights into causal and mechanistic relationships between the microbiota and host health.

Both as part of their endogenous metabolism and in response to external stimuli (e.g., diet), virtually all bacteria produce bacterial membrane vesicles (bMVs) (14). These nanoparticles reflect the nature and activity of the bacterial producer strain and can disseminate bacteria-derived molecules throughout the host organism (15). Because bMVs mirror bacterial activity, vesicle profiling offers a promising approach to assess diet-induced functional shifts in the gut microbiome. Because bacterial membrane vesicles encapsulate nucleic acids, proteins, metabolites, and lipids (14), investigating their functional effects (i.e., how immune and epithelial cells respond to them) provides an intrinsic form of multi-omics analysis. Their biological activity would herein reflect the combined action of these diverse classes of bioactives, rather than any single molecular layer in isolation. Moreover, bMVs represent a major functional output of bacteria as components of the microbial secretome. Differentiating them from non-vesiculated secreted factors, the bMVs rigid and thermostable structure protects cargo from degradation (16, 17), facilitates translocation beyond the intestinal lumen into systemic circulation, and enables efficient delivery of bioactive cargo to host cells (15, 18). These bacterial nanoparticles can efficiently be isolated from feces to allow a comprehensive view on bacterial metabolism and eventual effects on the host (19, 20). Indeed, bMV signatures can distinguish IBD patients from healthy controls with greater accuracy than bacterial compositions alone (21). As a result, gut-bMVs are increasingly studied as active biomarkers in metabolic, neurological, and inflammatory disorders (22).

Besides medication and physical exercise, strategies aimed at modulation of the gut-microbiota for the sake of host health improvement include dietary interventions and nutritional/neutraceutical supplementation. In recent years, rising attention is given to strategies aimed at direct modulation of the bacterial microbiota. Probiotics can for example stimulate the proliferation of bacteria considered benign such as *Bifidobacterium* and *Lactobacillus* (23) at cost of pathobionts such as *Escherichia/Shigella* and *Bilophila* (24–26), as determined by (sequencing-based) composition analysis. Moreover, expression of virulence factors by pathobionts such as *Pseudomonas aeruginosa* (27) and *Escherichia coli* (28) can be inhibited by probiotic bacteria. This illustrates how an intervention could not only act by diminishing (relative) abundance of a pathobiont, but also by inhibiting its disease-causing metabolisms. Next to the ingestion of live microbes, dietary macronutrients and the ratio to which they are ingested, can profoundly influence composition and activity of the microbiota as well. The dietary macronutrients fiber, protein, and fat can (i) act as substrate for microbial metabolism, (ii) influence metabolite production and (iii) interact with host physiology by consolidating gut-barrier function and modulating immune receptor activity (29–32). The bMVs produced in the human intestine reflect differential bacterial metabolism (14, 33) and mitigate (immune-)signaling between bacteria and to the human host organism (34). In this mini review we discuss means by which dietary modulation of the microbiome, may modulate bMV-mediated inflammatory signaling.

## Microbiota-related inflammation

A variety of external factors can modulate the composition and activity of the gut microbiota, which in turn alters inflammatory and immunological processes in their human host. Disturbances of the microbiota, characterized by loss of beneficial taxa at expansion of pathobionts could trigger inflammation, for example through increased endogenous production of LPS (6, 7, 35). Through its lipid anchor, LPS is inserted into bacterial membranes and as such, it is readily found on bMVs of Gram-negative bacterial origin, facilitating toxin delivery and presentation (36). Aside from LPS for Gram-negative bacteria derived vesicles, the bMV bioactive nature is reflected through carriage of other pathogen recognition receptor (PRR) agonists as well. These include peptidoglycan (PGN, Gram-negative and Gram-positive), lipoteichoic acid (LTA, Gram-positive) or bacterial DNA/RNA, protein and bacterial metabolites (14, 36–40).

bMV-mediated inflammatory signaling can be shaped by shifts in bacterial growth and metabolism. Dietary macronutrients, like fiber, protein and fat, are well established drivers of microbial metabolite production, thereby influencing inflammatory signaling in the host (26, 41). Fermentation of dietary fibers yields short-chain fatty acids (SCFA), including acetate, propionate and butyrate, which support intestinal epithelial health and suppress proinflammatory signaling (42). In contrast, microbial degradation of dietary protein produces branched-chain fatty acids (BCFA), which exhibit pro-inflammatory effects and may negatively affect host metabolism (42). As components of the bacterial secretome, SCFA/BCFAs and bMVs can mediate immunomodulatory interactions with the host. Crucially, bMVs can transport a plethora of bioactive bacterial metabolites including but not limited to amino acids, carbohydrates, cofactors, lipids and nucleotides (43). This renders them able to convey bacterial produce, and eventual diet-induced shifts in bacterial metabolism directly to the host’s immune system. As an emerging field, limited literature on gut microbiota–derived vesicles in relation to host metabolism is currently available, particularly when compared with well-established microbial metabolites such as SCFAs. However, increasing evidence suggests they might be involved in a wide range of microbe–host interactions.

## Diet-induced effects on bMV

### High protein diet

Diets enriched in specific macronutrients (protein, fat, fiber) can reshape which microbes make vesicles, what those vesicles carry, and how they signal to immune cells. A high-protein diet has been described to induce a microbial shift toward proteolytic and succinate-oriented metabolism in mice, accompanied by an increase in both the abundance and the inflammatory profile of gut-derived bMVs (44). Here, the elevated gut-luminal protein availability promotes this succinate production by specific gut bacteria, which is concomitant to enhanced bacterial production of reactive oxygen species and vesicles. *In vitro*, these high-protein diet induced bMVs exhibited increased ability to activate Toll-like receptor 4 (TLR4), which may support the increased secretory IgA responses observed *in vivo*. Importantly, gut-bMVs derived from a high-protein diet were reported to be more immunogenic than those derived from high-fat or high-carbohydrate diets, although no further investigation on the specific effects of macronutrient quality (e.g., animal or plant-based protein) was undertaken (44).

Dietary protein-derived metabolites may additionally modulate gut-bMV properties, as *in vitro* supplementation with glycine increased vesicle production by the probiotic *E. coli* Nissle 1917, whilst reducing vesicle LPS content (45). Moreover, the increase in overall gut-bMV production due to high-protein diets in mice increased markers of liver inflammation (44, 46). *In vitro* stimulation of hepatocytes with these gut-bMVs could reproduce this phenotype, and authors postulate that this occurs through delivery of bioactive intravesicular cargo (46). In this study however, the nature of this vesicle cargo is not investigated. As such, the specific bacterial sources and vesicular components responsible for these effects on the host remain to be identified. Notably, that the source and digestibility of the dietary proteins are pivotal, as different proteolytic fermentation products yield different metabolites (e.g., ammonia, amino acids and/or BCFAs) that too, would affect colonic bMV biogenesis and thus bMV immunogenicity. Summarizing, dietary protein is a pivotal driver of gut microbiota activity, and fiber-unbalanced high-protein intake may impose inflammatory pressures on the host. Nevertheless, means by which immunological properties of gut-bacterial membrane vesicles are modulated in response to high-protein diet are currently underexplored in humans.

### High fiber diet

Dietary fiber fermentation substantially shapes the gut metabolic environment by increasing luminal concentrations of short-chain fatty acids, which supports epithelial integrity, modulate immune responses, and influence systemic metabolism (42). Fiber-rich diets also shift the microbial community toward SCFA-producing taxa, impacting host-immunology (47), although what this means for bMV production by these taxa remains understudied. Importantly, bMVs readily contain fiber-degrading glycosidases (38, 39) whose expression is upregulated following a high-fiber diet. Such regulated processes hint toward an active bacterial membrane vesiculogenic response to diet which would contribute to a change in gut-derived bMV nature and, consequently, immunological properties. In pigs, *β*-mannan fiber administration yielded bMVs enriched for protein derived from Bacilli, Clostridiales and Enterobacteriales (48). This evidenced that dietary fiber administration could directly modulate the composition and, likely, inflammatory properties of gut-bacterial membrane vesicles.

Thus, implications of dietary fiber ingestion reach far beyond increasing gut-luminal SCFA levels as it reshapes the gut microbial ecosystem and may profoundly steer bMV production and properties. Nuances affecting this response (e.g., fiber type and quality), remain understudied in humans.

### High fat diet

Since dietary fats are primarily absorbed in the small intestine and therefore rarely reach the colon, they typically do not serve as an energy source for gut bacteria. However, in cases of fat malabsorption, high-fat intake, bile acid deficiencies or a combination of these factors, lipids can reach the colon and interact with the resident microbiota (49). This interaction can affect gut-microbial composition and functionality, thereby influencing bMV biogenesis. Fatty acids, in particular those of larger carbon chain length, exhibit antibacterial action through lysis and solubilization of bacterial cell membranes (50, 51). This suggests a mechanism through which dietary fats could modulate gut-bMV biogenesis through bacterial membrane destabilization.

In mice, a seminal study reported that a high-fat diet induced profound changes in gut-microbial bMV repertoires, including alterations in vesicle size, composition, and immunogenicity (52). Compared to regular chow diets, the high-fat diet relatively increased vesicle-associated LPS at the expense of lipoteichoic acid (LTA), which coincided with a higher prevalence of *Pseudomonas panacis* signatures in vesicle repertoires (52). This underlines further how ratios of dietary macronutrients in diets (e.g., high fat, low fiber) can drive immunological properties of bMVs by modulating the bacterial microbiota. Strikingly, here the effects of dietary intervention were more pronounced on bMV composition than they were on the bacteria themselves (52). This underlines the importance of investigating bacterial activity rather than bacterial abundance. Host-derived bile acids, secreted in response to lipid intake, can further modulate bMV production. Increased vesiculation has been observed in *Bacteroides fragilis* following exposure to host-derived bile salts (53). Finally, dietary effects on microbial vesiculation appear to be species-specific, adding another layer of complexity. For example, supplementation with saturated fatty acids induced vesiculation in *Bacteroides fragilis* but not in *Bacteroides thetaiotaomicron* (54). This illustrates that accurate phenotyping would require species-level resolution of microbiota-vesicle production. Further study of gut-bacterial vesicle production in relation to dietary fat intake (and macronutrient quality/type) is warranted in light of the ongoing obesity-epidemic, and its associated inflammatory comorbidities.

## Discussion—nutrient control of bacterial membrane vesicle-immune crosstalk

Significant health improvement can be achieved through diets that emphasize diverse, balanced and minimally processed foods. Such diets modulate the host microbiome and its activity, and suppress eventual gut-borne inflammatory signaling that could contribute to various pathologies. Bacterial membrane vesicles represent a summation of bacterial activity due to the many types of cargo they represent and disseminate. Studying vesicles produced by the bacterial microbiota therefore allows a holistic insight into bacterial metabolism, and how it interacts with its environment under change.

Dietary components not falling under one of the three macronutrients could profoundly influence the microbiota’s vesicle production as well. *In vitro*, a deficiency in availability of micronutrient iron has been found to decrease *E. coli* bMV production (55) and alter bMV cargo (56). Micronutrients with antioxidant properties such as vitamins C and E could potentially decrease bMV production in Gram-negative bacteria, as oxidative stress has been described as a trigger for vesicle formation by *P. aeruginosa* (57). Common plant-derived polyphenolic antioxidants such as quercetin could potentially increase vesiculation however, as their presence can cause membrane stress in *E. coli* and *Salmonella* (58). Alimentary supplementation of quercetin lead to a decrease in the relative abundance of these taxa in the cecal content of chickens (58). Importantly, *in vitro* supplementation of *Staphylococcus aureus* broth with Green-tea derived epigallocatechin gallate and citrus-fruit derived nobiletin yielded bMV repertoires with diminished ability to induce inflammation in immortalized human keratinocytes (59). Such examples illustrate how dietary micronutrients could influence both numbers and properties of gut-bacterial derived vesicles, with potential effects on host inflammatory signaling. Nevertheless, effects may be micronutrient- and strain-specific, and controlled human intervention studies are required to establish mechanistic evidence. Lastly probiotics, live micro-organisms ingested for their benign qualities, could (i) produce vesicles adding to endogenous gut-bMV repertoires and (ii) interact with other bacteria to influence vesicle production/cargo in endogenous microbes. For example, vesicles produced by probiotic *Lacticaseibacillus rhamnosus* and *A. muciniphila* promote anti-inflammation following oral administration in mice (60). Evidence from human intervention studies is scarce, but in healthy Korean adults, a kimchi-derived synbiotic beverage exerted stronger effects on gut-bMV repertoires than on bacterial community composition. Here, following 4 weeks of daily synbiotic intake a significant increase in bMVs by *Bifidobacterium* was observed, whilst the proportion of Proteobacteria bMVs was diminished (61).

The relevance of studying the microbiome’s interaction with its environment arises from the many diseases in which gut-dysbiosis plays a role. For example in T2D, the activity and vesicle production by *Akkermansia muciniphila* has been found lower than in healthy controls (62), suggesting that the decreased ability of this bacterium to produce vesicles, or the decreased presence of these vesicles could relate to disease pathophysiology. The precise ways in which *Akkermansia*-derived bMVs might confer eventual benign effects of the commensal bacteria remain to be elucidated. Exemplifying the complexity of the potential interaction of one microbe’s vesicles with the host; mass-spectrometry analysis of *A. muciniphila* derived vesicles previously identified 850 proteins associated with- or contained within these vesicles (63). Mechanistically, host responses to bacteria-derived biologicals typically result in altered inflammatory signaling, and novel immunomodulatory bacterial products which are likely to be expressed on bMVs continue to be described (64). Therapeutic modulation of certain gut-bMV repertoires through diet appears viable and safe in mice; *B. fragilis* vesicles can promote anti-inflammation in a mouse model for colitis (65). Notably, saturated fatty acid exposure has been described as a trigger for vesicle production by this taxon (54). Moreover, the aforementioned increased vesicle production by commensal *E. coli* Nissle 1917 under glycine exposure (45) could facilitate body weight modulation and improvement of glucose homeostasis, through its vesicle production in a diabetic mouse model (66).

**Figure 1:**
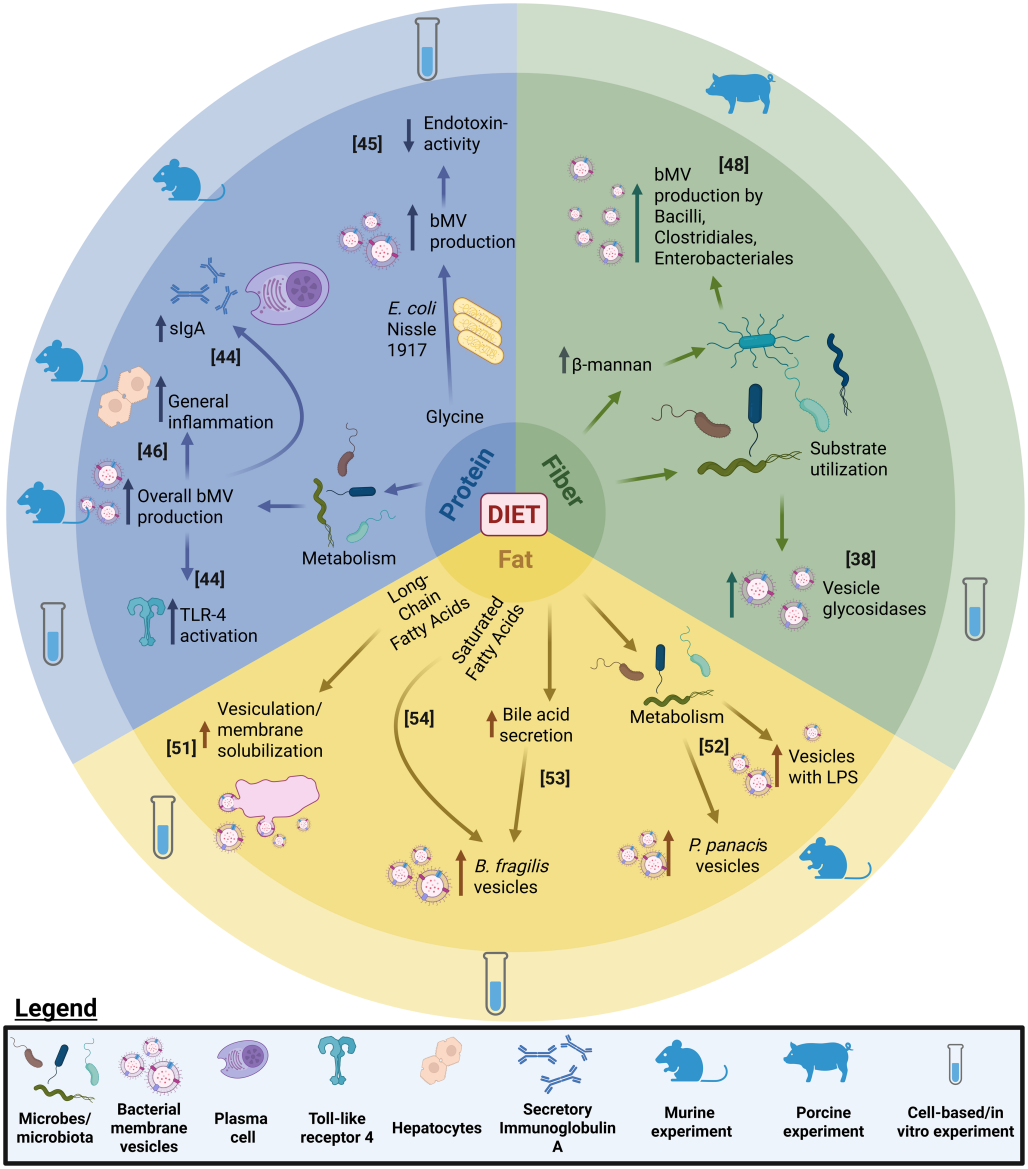
Schematic overview of the discussed effects of the dietary macronutrients protein, fiber, and fat on bacterial vesiculation production by microbes. Per experimental model, observed downstream effects of the modulation on vesiculation are depicted. bMV, bacterial membrane vesicle; TLR, Toll-like receptor; sIgA, secretory Immunoglobulin A. Numbers in brackets indicate concerning citation numbers in bibliography.

Human intervention studies investigating dietary effects on the microbiome, inflammation and host health are available (41, 67, 68). Nevertheless, studies on bMVs as holistic proxies of bacterial activity that mediate inter-Kingdom crosstalk are still limited. Emerging insights into microbe-host interactions suggest that gut-microbial compositionality (who is there) analyses do not predict functionality (what are they doing/producing) with high fidelity (8, 69). Investigating gut-bMV repertoires in response to dietary changes could provide added mechanistic understanding of intervention outcome. An overview of discussed works on modulation of bacterial membrane vesicle production through dietary macronutrients, and downstream effects is provided in Figure 1. It is important to note however, that bMV purification from feces or cecal content is laborious and lacks standardization. Their obtainment involves sequential steps generally involving centrifugation and filtration for contaminant removal, followed by vesicle enrichment through density gradient centrifugation or size exclusion chromatography (70, 71). As such, purity, quality and quantity of bMVs obtained is subject to the quality of the original sample and the purification procedure used. Importantly, feces-derived vesicle repertoires might be subject to the presence of eukaryotic vesicles (72), produced for example by cells lining the gastrointestinal epithelium tract. Elaborate protocols employing density-gradient centrifugation are available, to enrich bMVs from bodily fluids also containing eukaryotic vesicles (71). Although bacteria-specific bMV characterization methods (e.g., 16S rRNA sequencing, LPS-quantification) will not detect host EVs, aspecific methods such as nanoparticle tracking analysis do not allow discriminating vesicle types. Indeed, the presence of mammalian EV-specific tetraspanins (CD9/CD63/CD81) was detectable in feces-derived vesicle repertoires, albeit in minor magnitude compared to bMV-specific LPS and LTA (72). Moreover, much characterization of bMVs and the process of bacterial vesiculation is conducted using bacterial monoculture. However, vesicles produced by *E. coli* in the intestine arguably differ in properties and cargo from those originating from bacterial monoculture, as vesiculation is a consequence of bacteria interacting with their environment (14). Moreover, vesicle characterization results (e.g., vesicle size distributions by differential light scattering/DLS versus those by nanoparticle tracking analysis/NTA) are not interchangeable as various methods measure different aspects (70). Lastly, (functional) characterization of bMVs is typically performed using vesicles normalized by protein content (15, 16, 38, 39, 48, 52, 62) or particle counts (15, 18, 37, 46), however, neither strategy necessarily reflects physiological *in vivo* conditions per se. Approximating host-exposure conditions could be considered a more faithful approach reflecting in vivo vesicle fluxes, by for example employing donor-input based normalization strategies (e.g., all bMVs derived per mg of fecal matter). Nevertheless, experimental approaches should be tailored to the research question being addressed.

In conclusion, the vast gut-bacterial repertoire produces a plethora of molecules that are sensed by the host’s immune system, eliciting a wide range of immunological and physiological responses. Many of these molecules are reflected within- and on bMVs, whose nature and production can be modulated through diet. Diets supporting benign bacterial outgrowth would typically result in net increased production of bMVs and health-conferring produce by these taxa. bMVs serve as comprehensive carriers of bacterial molecular output and facilitate cargo delivery and dissemination. As such, they offer a powerful readout for investigating microbiota–host interactions in response to diet, although rigorous purification and standardized characterization are pivotal.
